# Self-reactive IgE and anti-IgE therapy in autoimmune diseases

**DOI:** 10.3389/fphar.2023.1112917

**Published:** 2023-01-23

**Authors:** Anna Olewicz-Gawlik, Arleta Kowala-Piaskowska

**Affiliations:** ^1^ Department of Immunology, Poznan University of Medical Sciences, Poznan, Poland; ^2^ Department of Infectious Diseases, Hepatology and Acquired Immunodeficiencies, Poznan University of Medical Sciences, Poznan, Poland

**Keywords:** Immunoglobulin E, anti-IgE, omalizumab, autoimmunity, autoimmune disease

## Abstract

Growing evidence indicates the pathogenic role of autoreactive IgE in autoimmune diseases. Incidence of autoimmune and allergic diseases in the industrialized countries is consistently icreasing, thus leading to concerted efforts to comprehend the regulation of IgE-mediated mechanisms. The first reports of a presence of IgE autoantibodies in patients with autoimmune diseases have been published a long time ago, and it is now recognized that self-reactive IgE can mediate inflammatory response in bullous pemhigoid, systemic lupus erythematosus, chronic urticaria, and atopic dermatitis. The advances in understanding the pathomechanisms of these disorders brought to a successful use of anti-IgE strategies in their management. The present review discusses the current state of knowledge on the IgE-mediated autoimmunity and anti-IgE treatment, and pave the way for further exploration of the subject.

## Introduction

The importance of immunotolerance was recognized more than a half century ago and the groundbreaking studies were awarded with the Nobel Prize in Physiology or Medicine in 1960 for Macfarlane Burnet. Since then our understanding of immune system and the mechanisms that lead to autoimmunity has increased significantly. It is now known that the distinction between self and foreign is not absolute, and that it depends not only on genetic predisposition and biological sex but could be influenced by many environmental factors.

Autoimmune diseases are not uncommon, with estimated prevalence of 7.6–9.4% (Cooper et al., 2009) and predominance in women (Fernandez Lahore et al., 2021). Based on the tissue involved, these diseases can be classified as organ-specific, such as type I diabetes mellitus (T1DM), multiple sclerosis (MS), autoimmune thyroiditis (AITD), bullous pemphigoid (BP), and systemic, like systemic lupus erythematosus (SLE), rheumatoid arthritis (RA), or systemic sclerosis, and the inflammatory reaction in many instances is mediated by autoantibodies (Theofilopoulos et al., 2017). Hundreds of loci associated with most of the autoimmune diseases have been identified, including certain MHC haplotypes and several other genes (Theofilopoulos et al., 2017). One can not underestimated the external exposome, which is believed to account for the increasing incidence of autoimmune and allergic diseases in the industrialized countries (Akdis, 2021; Celebi Sozener et al., 2022).

Many autoimmune diseases are characterized by the production of autoantibodies. Human B cells express five immunoglobulin isotypes: IgM, IgD, IgG, IgA, and IgE. Generally, a classic B-cell response starts with the secretion of the IgM, followed by isotype switching and production of IgG, IgA, or IgE. Antibody (Ab) isotype switch is regulated by costimulatory signals provided by follicular T cells, cytokine milieu, and the nature of antigen (Ag) itself (McHeyzer-Williams et al., 2009). The autoimmune processess are mainly mediated by IgG and IgM autoantibodies, however, it is apparent that autoreactive IgE is also present in patients with autoimmune conditions (Suurmond and Diamond, 2015). The mechanisms that lead in some B cells to a preference of IgE isotype switch for autoantibody production are still unclear. One of the possible theories of generating autoantibodies of different isotypes emphasizes the importance of post-translational protein modifications, such as phosphorylation, acetylation or carbamylation in creating neo-epitopes and contributing to the generation of autoantibodies (van Stipdonk et al., 1998; Zimina et al., 2008; Monahan et al., 2022).

### IgE biology

Our knowledge of immunoglobulin E (IgE) has travelled a long way since the discovery of IgE by Kimishige Ishizaka and his wife and research partner Teruko Ishizaka in 1966 (Ishizaka et al., 1950), to understanding the role of IgE in the development of autoimmunity. Conventionally, IgE is perceived as a mediator of immediate, allergen-specific immune response, known as type 1 hypersensitivity. However, the first reports of a presence of IgE antinuclear antibodies in patients with autoimmune diseases from the late 70s of the 20th century changed the image (Permin and Wiik, 1978). Shortly after Camussi and colleagues detected basophil sensitization by IgE antibodies to nuclear antigens and subsequent degranulation of these cells to the examined autoantigens (Camussi et al., 1982). As a consequence of these and further discoveries anti-IgE therapy has been introduced (Presta et al., 1993). Homology between autoantigens and exogenous allergens leading to cross-reaction of IgE particles, or primary break in self-tolerance leading to the production of IgE autoantibodies are among potential mechanisms of generation of autoreactive IgE (Charles, 2021). IgE directed against autoantigens were observed in a number of autoimmune conditions, including SLE (Atta et al., 2010), BP (van Beek et al., 2016), chronic spontaneous urticaria (CSU) (Panaszek et al., 2017), AITD (Guo et al., 1997), multiple sclerosis (Seals et al., 2022), and mixed connective tissue disease (Lamri et al., 2021).

### IgE-receptors and IgεR-bearing cells

There are two types of receptors for IgE: the high-affinity receptor FcεRI expressed as a classic αβγ2 tetrameric form by mast cells and basophils but also in a trimeric form by macrophages, dendritic cells (DCs), eosinophils, platelets and neutrophils, and the lower affinity receptor, CD23, present on various cells including mature B cells, activated T cells, Langerhans cells, plasma cells, monocytes, eosinophils, and epithelial cells (Galli and Tsai, 2012). The FcεRI receptor is composed of an α-, β-, and two γ subunits and is a member of a family of related antigen/Fc receptors (Turner and Kinet, 1999). It is known, that basophils and mast cells promote Th2 responses by rapid IL-4 production subsequent to crosslinking of their FcεRI through preexisting Ag-IgE complexes (Khodoun et al., 2004). Moreover, basophils cooperate with DCs in mounting an inflammatory response (Tang et al., 2010). In this place it is worth noting, that the trimeric variant of FcεRI present on plasmacytoid DCs (pDCs) is involved in counterregulation of type I interferon (IFN) release in response to Toll-like receptor (TLR)-7 and TLR-9 stimulation (Henault et al., 2016). The discovery of soluble FcεRI in human sera in 2011, which competed with membrane-bound FcεRI to bind IgE, resulted in hypothesis about the regulatory mechanism of IgE-mediated cell activation (Dehlink et al., 2011). The second type of IgE receptor, CD23, belongs to the family of C-type lectins, and contain three lectin domains located on the C-terminal extracellular end of the molecule, and beside IgE it has also binding site for CD21, thus regulating IgE synthesis (Hibbert et al., 2005). While interaction of IgE-Ag complexes with FcεRI elicits cellular degranulation process and accounts for immediate hypersensitivity reactions and the induction of clinical symptoms of allergy, the binding between IgE and CD23 has been reported to be involved in Ag presentation, and the transport of antigens across airway and intestinal epithelial barriers (Gasser et al., 2020). Furthermore, beside being a receptor for IgE and CD21, CD23 can also be released from cell surfaces as a range of freely soluble (s) CD23 proteins (Acharya et al., 2010). Soluble CD23 levels were found to be elevated in a range of autoimmue diseases, including Sjögren’s syndrome, SLE (Bansal et al., 1992), and RA (Bansal et al., 1994). Classically, IgE does not have the capacity to activate complement through the classical pathway, but it was shown that human monoclonal IgE activated the classical pathway of complement (Saint-Remy, 1984), and that aggregated human monoclonal immunoglobulins of IgE class are able to activate complement through the alternative pathway (Basta and Szebeni, 2004).

### Anti-IgE treatment

Until today, the only anti-IgE therapy approved for clinical use is omalizumab, a recombinant humanized monoclonal antibody. Omalizumab, the humanized recombinant monoclonal antibody against IgE, is known to result in a marked reduction in serum-free IgE and the downregulation of IgE receptors on circulating basophils (Chularojanamontri et al., 2009). Clinical trials with another anti-IgE agent, high affinity anti-IgE monoclonal antibody, ligelizumab, are under way. Both drugs recognize a distinct IgE epitope, and the interaction of ligelizumab with IgE has been shown to be around 88‐fold stronger than the IgE binding of omalizumab (Gasser et al., 2020). In addition, omalizumab was shown to downregulate CD23, making cells less sensitive to allergens, and also inhibited binding of IgE to CD23 more potently than ligelizumab, thus potentially exerting greater effect on the inhibition of IgE-mediated Ag presentation and IgE-mediated transport across epithelial barriers (Gasser et al., 2020). On the other hand, ligelizumab was capable of binding and aggregating CD23:IgE complexes on the surface of B-cells, which has not been observed for omalizumab (Gasser et al., 2020). Moreover, ligelizumab displayed stronger capacity than omalizumab to block the binding of free IgE to FcεRI on human pDCs, thus resulting in a greater restoration of TLR9-ligand-induced IFN-α production (Benito-Villalvilla et al., 2022). All these differences between omalizumab and ligelizumab could be the reason for different effects of the drugs in IgE-mediated conditions. Third anti-IgE monoclonal antibody, quilizumab, is a humanized, afucosylated, monoclonal IgG1 antibody, which binds membrane IgE at the M1-prime segment, which is absent in soluble IgE (Brightbill et al., 2014). The limitation of the drug is the fact that it can not target IgE plasma cells because of their lack of membrane IgE, and there are no ongoing clinical trials with the agent. Another anti-IgE approach under investigation is UB-221. It is a humanized IgG1 monoclonal antobody (clone 8D6) that targets the Cε3 domain of IgE antibody, and similarly to omalizumab, it can not bind to IgE bound by the FcɛRI on basophils and mast cells (Shiung et al., 2012). However, UB-221 has the capacity to bind to IgE bound by CD23 (Shiung et al., 2012; Kuo et al., 2022), what makes it a significant CD23-mediated IgE synthesis downregulator.

## Diseases where self-reactive IgE is known to have a pathogenic role

### Systemic lupus erythematosus

SLE is a chronic, multisystem, inflammatory disease of autoimmune pathogenesis. The abnormal activation of immune response is characterized by activation of type I IFN system and the production of autoantibodies, mainly against nuclear antigens (Kirou et al., 2005). Although until recently the majority of pathogenic autoantibodies in SLE was considered to be of the IgG class (Villalta et al., 2013; Dema and Charles, 2016), the study by Charles et al. showed that the presence of autoreactive IgE in SLE subjects was also associated with increased basophil activation and enhanced disease activity (Charles et al., 2010). In addition, Dema et al. observed delayed SLE development and severity associated with IgE deficiency (Dema et al., 2014a). Moreover, activation of basophils by autoreactive IgE resulted in an amplification of autoantibody production and IL-17 secretion, thereby contributing to the SLE pathogenesis (Pan et al., 2017). While in patients with allergic diseases IgE is directed against allergens, in patients with SLE IgE autoantibodies react mainly with nucleic antigens and are capable of triggering IFN-mediated responses through TLR7 and TLR9, while IgE against allergens lacks this property (Sanjuan et al., 2016). In a study of 92 SLE patients, the immunoblotting of sera obtained from 29 patients positive for antinuclear IgE antibodies revealed IgE reaction with nucleosomes (79.3%), dsDNA (48.3%), SS-A/Ro (48.3%), SS-B/La (18.7%), Sm (48.3%) and RNP (62.1%) (Atta et al., 2010). In a multicentre study of 196 SLE patients, approximately 65% had IgE antibodies to the seven autoantigens tested, and the positivity increased to almost 83% when only those subjects with active disease were considered (total vs. active disease: dsDNA 35,4% vs. 62%, Sm 7.5% vs. 6.1%, SS-A/Ro 8.5% vs. 4.1%, SS-B/La 4.1% vs. 0,2%) (Dema et al., 2014b). Furthermore, autoreactive IgE to certain antigens showed a highly significant association with hypocomplementemia, suggesting potential role of these autoantibodies in lupus pathogenesis (Dema et al., 2014b). Interestingly, in the study no association of IgE levels with gender or ethnicity were found and no correlation between the total levels of IgE and the levels of IgE autoantibodies were observed. Henault and colleagues reported that in SLE subjects IgE antibodies specific for dsDNA activated pDCs, which are known to be involved in a viral defense, by binding cell surface FcɛRI, what led to the secretion of substantial amounts of IFN-α in a TLR9-dependent manner (Henault et al., 2016). It is worth to note that pDCs produce 200 to 1000 times more IFN than other blood cells (Siegal et al., 1999), and INF-α has been found to inhibit degranulation of eosinophils, histamine release in mast cells, prevent IL-3-mediated priming of basophils, and reduce allergic responses by preventing GATA3 from enhancing its own expression in T cells, thus in patients with SLE with IgE antibodies allergies may not be common (Sanjuan et al., 2016). Of note, Guo et al. observed the adverse impact of co-existing atopy on the severity and prognosis of juvenile SLE (Guo et al., 2017). Regarding further association between IgE and autoimmunity, Sekigawa and colleagues found that the serum IgE level seems to be an important factor in the occurrence of foetal loss in mothers with anti-SSA antibody detected by ELISA (Sekigawa et al., 2004). IgE was also found to exert an immunoregulatory role in the inflammatory response in SLE by promoting immune cell recruitment to secondary lymphoid organs (Dema et al., 2014a). Further, Khoryati and colleagues observed significant inverse correlation of IgE levels with SLE activity, and IgE levels were not associated with the presence of antinuclear IgE (Khoryati et al., 2016). Moreover, IgE treatment down-regulated expression of TLR-9 and IFN regulatory factor 7 in pDCs and decreased IFNα secretion upon stimulation with TLR-7 and TLR-9 ligands (Khoryati et al., 2016). Finally, in 2019 the results of a randomized clinical trial of the safety, tolerability and clinical efficacy of omalizumab in SLE have been published (Hasni et al., 2019). Omalizumab was well tolerated with no allergic reactions, and the treatment led to a trend towards reduction in IFN gene signature, especially in subjects with high baseline IFN signature. The results of the study are in agreement with the recent discovery that omalizumab therapy reconstitutes Treg homeostasis in vitro by restoring the ability of human pDC to induce Foxp3^+^Tregs (López-Abente et al., 2021). However, the clinical trial included a small number of SLE patients with mild clinical disease with no organ threatening manifestations, and the promising results need to be confirmed in a larger cohort with more active disease.

### Bullous pemphigoid

Bullous pemphigoid belongs to a group of subepidermal autoimmune blistering diseases, mainly affects elderly persons over 65 years of age, and is characterized by damage to the protein constituents of the basement membrane zone (BMZ). The incidence of BP is 6 times higher in North America and Europe when compared to Asia (Lu et al., 2022). The pathogenesis of BP is associated with the presence of autoantibodies directed againts hemidesmosomal proteins: BP antigen 2 (BPAG2/BP180, a transmembrane glycoprotein, type VII collagen) and the intracellular BP antigen 1 (BPAG1/BP230) located on the BMZ in the skin and mucous membranes (Kayani and Aslam, 2017). Autoreactive IgE directed against BP180 target mainly the extracellular noncollagenous (NC) 16A domain of BP180 (BP180 NC16A) (Dresow et al., 2009). It was observed that phosphorylation of the NC16A domain may contribute to the generation of autoantibodies against this region and thereby influence the pathogenesis of BP (Zimina et al., 2008). Although autoantibody isotypes against BP180 and BP230 are usually of IgG class, the role of IgE isotype has been also implicated (Kayani and Aslam, 2017), and both classes of immunoglobulins coexisted in BP patients, with levels increasing along with disease severity (Sanjuan et al., 2016). In a study by Dimson et al. elevated IgE levels were found in 70% of untreated BP patients, and BP180-stimulated histamine release was significantly higher in basophils obtained from untreated BP patients, what implicated functionality of IgE autoantibodies (Dimson et al., 2003). The results of Freire and colleagues showed that circulating IgE was not only elevated, but also specific for BP180 and BP230 and that self-reactive IgE and IgG recognized the same epitopes in the BP180 molecule. Furthermore, in the skin of patients with BP, IgE reacted with FcεRI on mast cells and eosinophils, and was colocalized with the shed fragments of BP180. In addition, immune complexes of IgE-BP180 had capability to cross-link FcεRI and cause the degranulation of basophils (Freire et al., 2017). Interestingly, IgE anti-BP230 serum levels were strongly associated with local eosinophil accumulation (Ishiura et al., 2008). The mechanisms that attract FcεRI-bearing eosinophils to the BMZ, although not fully explained, are based on a sequence of events, starting with mast cells alternation, Langerhans cells activation, and the formation of immune cell infiltration, first with lymphocytes, later with eosinophils and basophils (Dvorak et al., 1982). Briefly, in response to the signals provided by mast cells and the presence of IgE, eosinophils accumulate and degranulate in the skin, triggering production of further chemotactic and inflammatory mediators by keratinocytes, thus leading to the positive feedback loop of eosinophil recruitment (Amber et al., 2018). The pathogenic role of IgE in BP was further confirmed by successful treatment of BP with omalizumab (Fairley et al., 2009; Dufour et al., 2012; London et al., 2012; Yalcin et al., 2014; Lonowski et al., 2020). In a recent retrospective analysis of BP patients who presented with corticosteroid-dependent disease along with contraindications to the use of other immunosuppressive treatments, omalizumab therapy was associated with a reduction in the dose of systemic glucocorticosteroids and complete resolution of skin lesions in 100% of the patients (Vassallo et al., 2022). A reduction of pruritus and circulating IgE was found in 40% of patients, anti-BP180 and BP230 IgGs were decreased in 60% and eosinophils in 80% (Vassallo et al., 2022). The only study on ligelizumab in BP (NCT01688882) has been terminated because the predefined criteria of efficacy were not reached.

### Atopic dermatitis

Atopic dermatitis (AD) is a chronic, non-infectious inflammatory disease of the skin of multifactoral etiology, including environmental factors, impaired skin barrier function, and immune system dysregulation (Otsuka et al., 2017). The pathogenesis of inflammation in AD is highly complex, with remissions and relapses and a heterogeneous clinical course strikingly resembles autoimmune diseases (Floca et al., 2022). AD typically occurs early in life in a large majority of cases, with 50% of AD cases beginning in the first year of life, and 85% beginning by the age of five (Sacotte and Silverberg, 2018). It affects 15–20% of children and 1–3% of adults worldwide, and the incidence of AD has increased by 2- to 3-fold during the past decades in developed countries (Nutten, 2015). In brief, in AD Langerhans cells stimulate a Th2 cell response by presenting IgE-bound antigens (Mudde et al., 1990). Excessive generation of Th2 lymphocytes leads to increased production of IL-4, IL-5 and IL-13, and stimulates eosinophils and IgE antibodies (Matsunaga and Yamauchi, 2016). As reviewed by Tang and colleagues, increased IgE levels and specific IgE in plasma were observed in AD patients, and the total IgE levels strongly correlated with the prevalence of IgE autoreactivity (Tang et al., 2012). Also AD severity has been associated with autoreactive IgE (Kinaciyan et al., 2002; Pellefigues, 2020). Furthermore, Zeller et al. demonstrated that at least 140 IgE-binding self-antigens is associated with AD (Zeller et al., 2009). Their data indicated that IgE-mediated autoreactivity was confined to patients with AD, and from 8.7% to 29% out of AD patients had self-reactive IgE for different autoantigens, including actin-α, tubulin-α, eukaryotic translation initiation factor 6 (eIF6), HLA-DR-α, and microtubule-associated protein RP/EB family member 2 (MAPRE2) (Zeller et al., 2009). Finally, Holmes and colleagues reviewed 14 studies on the presence of specific IgE autoantibodies in AD (Holmes et al., 2019). The results of the analysis indicated that there is an association between autoreactivity with regard to autoantibodies of IgE class and AD, as well as disease severity in AD (Holmes et al., 2019). An association between AD and susceptibility to autoimmune diseases was confirmed by a study on 8112 adult patients with AD and age and gender matched controls, and the significantly higher occurrence of autoimmune comorbidities in smoking AD patients was also noted (Andersen et al., 2017). According to the data of Mothes et al., early infancy is considered the critical period for IgE autosensitization (Mothes et al., 2005). Based on the data from two recent systematic reviews on the use of omalizumab in AD it can be concluded that omalizumab was safe and well-tolerated in AD patients and the beneficial effect of the treatment was achieved by 43% - 62.5% of AD patients (Wang et al., 2016; Holm et al., 2017). In 2020 the results of the Atopic Dermatitis Anti-IgE Pediatric Trial (ADAPT) assessing the effect of omalizumab in children with severe AD were published. It was found that that omalizumab use improved disease severity and patient quality of life (Chan et al., 2020). Efficacy of omalizumab in AD was also confirmed in case reports (Alzyoud, 2022). However, future larger clinical trials are needed to fully support the evidence from previous studies and further clarify the role of anti-IgE therapy in AD.

### Chronic spontaneous urticaria

According to the 2022 European Academy of Allergology and Clinical Immunology (EAACI) guidelines, urticaria is a condition characterized by the spontaneos development of wheals (also called hives), angioedema, or both, and is regarded as chronic if the above symptoms appearance due to known or unknown causes last more than 6 weeks (Zuberbier et al., 2022). CSU is considered to be primarily a mast cell-driven disease, as reviewed in Church et al. (Church et al., 2018), although eosinophils also emerged as an important player in CSU pathogenesis (Altrichter et al., 2020). An important role of autoimmunity in patients with urticaria was postulated for several decades based on the positivity of autologous serum skin test in some patients (Grattan et al., 1986). Puccetti and colleagues demonstrated the presence of autoantibodies against CD23 in a large percentage of CSU patients (Puccetti et al., 2005), autoantibodies directed to the alpha subunit of FcεRI has also been detected in a subpopulation of chronic urticaria (CU) patients (Kikuchi and Kaplan, 2001). Interestingly, autoreactive CD4+ T cells that target FcεRIα were detected in the majority of patients with CSU, and their cytokine secretion profile was more typical of a Th1 response (Auyeung et al., 2016). In 1999 Bar-Sela and colleagues described a case of CU associated with elevated IgE and the presence of anti-thyroid peroxidase (anti-TPO) antibody of IgE isotype (Bar-Sela et al., 1999), however the results of further studies investigating the presence of anti-TPO IgE in CU patients were contradictory, with the frequency of these antibodies ranging from 0% (Tedeschi et al., 2001) to 54,2% (Altrichter et al., 2011). Nevertheless, an association between CU and thyroid autoimmunity has been well-documented (Zauli et al., 2001; Cebeci et al., 2006), and confirmed by the results of the multicenter, randomized, double-blind, placebo-controlled study on the efficacy and safety of omalizumab in patients with CU and anti-TPO IgE antibodies (Maurer et al., 2011). In the study omalizumab proved to be an effective treatment option for the enrolled subjects who were refractory to conventional treatment (Maurer et al., 2011). The results of a large population study from Israel, encompassing the data of 12 778 patients diagnosed with CU during 17 years in a large health maintenance organization showed that female patients with CU had a significantly higher incidence of RA, Sjögren syndrome, celiac disease, T1DM, and SLE, which were diagnosed in the majority of patients during the 10 years after the diagnosis of CU (Confino-Cohen et al., 2012). Later, in the study using array analysis, Schmetzer et al. found more than 200 IgE autoantigens in patients with CSU that were not found in control subjects, with only IgE autoantibodies to IL-24 presented in all investigated patients. Further *ex vivo* experiments confirmed functionality of anti-IL-24 IgE autoantibodies (Schmetzer et al., 2018), and in patients with CSU treated with autologous serum therapy significant decrease of IgE-anti-IL-24 serum levels, but not IgG-anti-IL-24 serum levels, was observed in responders in contrast to non-responders (Yu et al., 2019). Nevertheless, additional studies are needed to determine the role of IL-24 and anti-IL-24 IgE in CSU as potential treatment targets. Contrary, strong evidence exists on behalf of omalizumab therapy in CSU. Phase 3 randomized studies confirmed the efficacy and safety of omalizumab in patients with CU (Kaplan et al., 2013; Maurer et al., 2013; Saini et al., 2015), and the anti-IgE therapy was approved for use in the population in 2014 in both the EU and the USA (Wu and Jabbar-Lopez, 2015). What’s more, the type of autoimmunity, IgE vs. IgG, might determine time to response to omalizumab treatment as was shown by Gericke et al. (Gericke et al., 2017). In a recent single-centre retrospective study of CU patients, comparing responders and non-responders to omalizumab treatment, the eosinophil count, basophil count, and total IgE level before omalizumab treatment were significantly higher in the group of responders to omalizumab treatment, and baseline anti-thyroglobulin (TG) antibodies were significantly higher in the group of non-responders (Cakmak, 2022), but the anti-TG antibodies were in the class G immunoglobulin. Moreover, the treatment effects of omalizumab in CSU showed dependence on the presence of autoantibodies to IgE or FcεRI, which highly correlated with basopenia and non-responder status (MacGlashan et al., 2021).

In a phase 2b core study in CSU patients (Maurer et al., 2019), which was followed by a 1-year phase 2b extension study (Maurer et al., 2022), ligelizumab showed a clear dose–response relationship with regard to complete hives response, rapid and sustained symptom control with higher frequency when compared to omalizumab, and was well-tolerated. The results of a recent phase 2b study have demonstrated that ligelizumab effectively reduced angioedema and urticaria symptoms, and improved health related quality of life in patients with moderate to severe CSU (Metz et al., 2022). Most recently two phase 3 clinical trials (NCT03580356 and NCT03580369) on the efficacy and safety of ligelizumab in the treatment of CSU in adolescents and adults, which together enrolled more than 2100 participants, have been completed. Their results are to be published.

Two more anti-IgE approaches were tested in CSU. The results of a randomized, double-blinded, placebo-controlled trial of quilizumab in adults with refractory CSU exhibited median reduction in serum IgE levels by approximately 30% over 20 week, but it did not cause clinically relevant improvements in CSU severity and activty scores (Harris et al., 2016). Another antibody, UB-221, administered as a single dose to patients with CSU in a first-in-human trial showed durable symptom relief concomitant with a rapid reduction in serum free-IgE level (Kuo et al., 2022).

### Hiper IgE syndromes

Hyper-IgE syndromes (HIES) are rare primary immunodeficiencies characterized by elevated serum IgE, eczema and recurrent infections most commonly affecting skin and lungs, with the mean prevalence of less than 1:1000000 (Gernez et al., 2018). The main two forms of this heterogeneous group of inborn errors of immunity are autosomal dominant (AD)-HIES variant and autosomal recessive (AR)-HIES variant. The AD-HIES is caused by loss-of-function mutations in signal transducer and activator of transcription 3 (STAT3) (Holland et al., 2007), while in patients with AR-HIES homozygous and heterozygous mutations in DOCK8 were identified (Engelhardt et al., 2009). Moreover, SLE-like autoimmunity with predominant renal involvement have been identified in a cohort of patients with AD-HIES (Goel et al., 2021). There are several reports on the use of omalizumab in HIES patients, mainly with recalcitrant skin lesions, with improvement of symptoms in all cases (Bard et al., 2008; Marcotte, 2008; Chularojanamontri et al., 2009; Akbaş et al., 2017; Alonso-Bello et al., 2019; Gomes et al., 2020; Lan et al., 2022), but to provide ample evidence further studies and long-term follow-up are necessary. Increased serum IgE levels can also be seen in other primary immunodeficiencies, the main of which are two immune disregulation syndromes, autoimmune lymphoproliferative syndrome (ALPS) and the immunodysregulation, polyendrocrinopathy and enteropathy X‐linked (IPEX) syndrome. Elevated IgE levels and eosinophilia were observed in 16%‐25% of patients with ALPS (Butt et al., 2015). Interestingly, the relative absence of allergic diseases among ALPS patients with high IgE levels suggests an association between IgE production and autoimmunity that is independent of allergy, and could be a result of Ag‐independent differentiation of unconventional germinal center B cells into IgE‐producing plasma cells (Butt et al., 2015). In IPEX, aside from elevated IgE, presence of eosinophilia and absence of CD4+CD25hiFOXP3+Tregs is observed (d’Hennezel et al., 2012). Elevated levels of IgE concomitant with autoimmunity were also described in CD3γ deficiency, but the specificities of these IgE were not investigated in the study (Gokturk et al., 2014). So far no reports on the use of anti-IgE treatment in ALPS and IPEX have been published, and the studies regarding IgE specificity and potential IgE autoreactivity in the above primary immunodeficiencies are lacking.

Table 1 summarizes the information about the presence of IgE autoantibodies and studies on anti-IgE treatment in autoimmune diseases.

**Table 1:** A summary of autoimmune diaseases mediated by autoreactive IgE and the application of anti-IgE therapy.

Autoimmune disease	IgE autoantigens	Anti-IgE treatment studies		Efficacy of anti-IgE therapy	
		omalizumab	ligelizumab	omalizumab	ligelizumab
Systemic lupus erythematosus Atta et al. (2010); Dema et al. (2014b)	dsDNA, Sm, SSA (Ro), SSB (La), nucleosomes, RNP	Yes	No data	Yes	n/a
Bullous pemphigoid Dimson et al. (2003); Dresow et al. (2009)	BP180, BP230	Yes	Yes	Yes	No
Chronic spontaneous urticaria Confino-Cohen et al. (2012); Schmetzer et al. (2018)	thyroid peroxidase, thyroglobulin, IL-24	Yes	Yes	Yes	Yes
Atopic dermatitis Zeller et al. (2009)	More than 140 autoantigens, including ribosomal P2 protein, cyclophilin, manganese superoxide dismutase, Hom s1, Hom s2, Hom s3, Hom s4, Hom s5	Yes	No data	Yes	n/a
Pemphigus vulgaris Nagel et al., (2010); Ji-Xu et al. (2022)	desmoglein 3	Yes	No data	Yes	n/a
Multiple sclerosis Mikol et al. (2006)	small myelin protein-derived peptides	No data	No data	n/a	n/a
Autoimmune thyroiditis Guo et al. (1997)	thyroid peroxidase	No data	No data	n/a	n/a
Systemic sclerosis Daskalova et al. (1997)	elastin	No data	No data	n/a	n/a
Mixed connective tissue disease Lamri et al. (2021)	U1 small nuclear ribonucleoprotein	No data	No data	n/a	n/a
Autoimmune uveitis Muiño et al. (1999)	retinal S antigen	No data	No data	n/a	n/a
Rheumatoid arthritis Permin and Wiik (1978)	Granulocyte antigens	No data	No data	n/a	n/a

### Open questions and future directions

The exact role of IgE in autoimmunity remains unclear, and it affects the decision on introducing the anti-IgE therapy. Fortunately, despite increased IgE levels in many autoimmune diseases the frequency of IgE mediated anaphylaxis is low (Butt et al., 2015; Sanjuan et al., 2016). The possible explanation for the phenomenon could be the differences in the affinity of IgE. It has been shown that high-affinity but not low-affinity IgE causes anaphylaxis (Wang et al., 2010; Croote et al., 2018), however the studies on IgE affinity in autoimmune conditions are lacking. Moreover, Xiong and colleagues reported that high-affinity IgE predominantly arise from IgG1 through the sequential switching pathway (Xiong et al., 2012), and it would be of interest to understand the nuances of IgE generation in autoimmunity. Although it is generally known that Ag-Ab complexes of different izotypes play an important role in the activation of the immune response in immune-mediated diseases, and the mechanism of action of IgG-Ag complexes are well-studied, still little is known about the function of immune complexes containing IgE. Recently a novel mechanism by which IgE-allergen complexes regulate allergic inflammation was described, depending on the proximity of IgE binding sites on the allergens (Gieras et al., 2016). Further studies on IgE-Ag complexes in autoimmune diseases are needed. Furthermore, the results of a recent study revealed that IgE glycosylation is absolutely necessary in allergic reactions (Shade et al., 2015), thus a question arouses, is IgE associated with autoimmunity also glycosylated, and if so, are there any differences in glycosylation between these two IgE entities? T follicular helper cells (Tfh) are a key extrinsic regulators of IgE as the major cellular source of IL-4 and IL-21, the main activating and inhibitory cytokines for IgE class switch recombination (Wade-Vallance and Allen, 2021). Aberrant expansion of Tfh cells was observed in AD, SLE, BP, and CU (Li et al., 2013; Szabó et al., 2017; Gao et al., 2020; Jin et al., 2022). These findings may contribute to the development of new targets for therapeutic interventions for these diseases. Which makes the issue even more complicated, there are reports existing on the development of autoimmune diseases in the course of omalizumab treatment (Rams et al., 2018; To et al., 2021). On the other hand, efficacy of omalizumab was observed also in patients with other autoimmune diseases, like eosinophilic granulomatosis with polyangiitis (Basta et al., 2020). Additional research on the role of IgE in the disorders conventionally perceived as non-IgE-mediated should further clarify their pathogenesis, and future studies on the effects of omalizumab and ligelizumab in autoimmune diseases are likely to shed some light on the mechanism of anti-IgE therapy in these diseases.
